# Evaluation of the Thermophysical, Sensory, and Microstructural Properties of Colombian Coastal Carimañola Obtained by Atmospheric and Vacuum Frying

**DOI:** 10.1155/2022/7251584

**Published:** 2022-06-07

**Authors:** Diofanor Acevedo C., Piedad M. Montero, Jhon Rodriguez Meza, R. Sandrith Sampayo, Raul Jose Martelo

**Affiliations:** ^1^Faculty of Economic Sciences, Tourism Administration Program, Innovation, Management and Engineering Research Group, Universidad de Cartagena, 130011, Colombia; ^2^Faculty of Engineering, Food Engineering, Agricultural and Agro-Industrial Innovation and Development Research Group IDAA, Universidad de Cartagena, 130011, Colombia; ^3^Faculty of Engineering, Communications and Informatics Technologies Research Group GIMATICA, Universidad de Cartagena, Colombia

## Abstract

The quality of fried products affects consumer purchase decisions, and frying is an important stage in the production process. The objective of this research was to evaluate the thermophysical properties, the sensory quality, and microstructure of Colombian coastal Carimañola traditionally manufactured, in atmospheric frying and vacuum frying conditions. Lower moisture and fat content were reported in samples fried under vacuum compared to samples fried under atmospheric conditions, which is associated with the vacuum pressure during the process. Thermophysical properties, related to heat transfer in the samples, showed a correlation between thermal conductivity and moisture content. The micrographs visualized the changes in the porous structure of the coastal Carimañola. A greater effect was evidenced in the samples obtained by atmospheric frying because higher temperatures were used. The sensory evaluation reflected a preference for Carimañolas made with conventional frying. This research provides a basis for consumer purchases of traditionally fried products made with vacuum frying.

## 1. Introduction

Cassava (*Manihot esculenta*) is considered an essential food in 105 countries, it is one of the most commercialized roots in the world, and its consumption provides a high amount of energy mainly as a source of carbohydrates [1]. In Colombia, this crop ranks fifth in terms of production, after sugar cane, banana, potato, and rice [2, 3]; it is cultivated in 32 departments, predominantly in the Caribbean region.

Carimañola is a fried product widely consumed in the Colombian Caribbean region, plays an important role in the culinary tradition, and is one of the main economic supports of many families in the region [4]. The main ingredient in Carimañolas is cassava dough; it is often stuffed with meat or cheese and then subjected to frying treatments. This type of product, mostly handmade, represents a way of taking advantage and transforming cassava into by-products with greater added value, since they are more attractive to consumers due to the sensory properties conferred in the frying process [5].

Frying is a very common cooking technique [6]; it consists of immersing food in oil at high temperatures, generally between 150°C and 180°C, depending on the raw material, generating physical and chemical changes [7], such as starch gelatinization [8], denatured proteins [9], and formation of crusts [10].

Frying is the most critical point in the production process of Carimañola; it includes heat and mass transfer processes that determine the characteristics of the final product. Initially, convection of heat occurs from the hot oil to the surface of the food, and then, this heat is conducted from the surface to the center of the product. In general, the frying process consists of 4 stages: first is heating from the moment the product is incorporated into the oil until the surface temperature of the food is equalized to the temperature of the oil by free convection; then, in the surface boiling stage, water vapor bubbles appear and generate turbulence in the surrounding oil and the heat transfer process changes to forced convection; in the third stage, the internal temperature of the food increases, the rate of moisture loss and bubbling is reduced, and it is here that starch gelatinization and protein denaturation occur. And finally, in the fourth stage, the appearance of bubbles ceases [11].

The study of the thermophysical properties of foods is an important step to optimize the rate of heat transfer in products; it is known that an increase in the rate of transfer could improve the overall efficiency of the process [12]. Furthermore, the analysis of thermal properties such as specific heat, thermal diffusivity, and thermal conductivity is a fundamental aspect of the study and design of heat transfer processes [13], equipment design, and improving product quality. In thermal treatments, these properties depend on the temperature applied and the chemical composition of the food matrix, in addition to allowing calculation of the amounts of heat and time required for processing each food [14]. Caro et al. [5] demonstrated that the characteristics of the coastal Carimañola are influenced by processing conditions, temperature, and frying time.

The unique sensory characteristics of fried foods are the main reason why they enjoy consumer preference, including fat content that improves texture and flavor [15]. The continuous increase in consumer awareness of healthy and safe fried products has led to research on these products [16, 17]. Thermal processes are important stages that improve the sensory acceptance of various foods that are needed for human nutrition. In the vast majority of cases, these thermal processes are related to traditions and the need for ready-to-eat food [18].

Fried products have a common characteristic, the so-called crunchy texture, which indicates the quality of the finished product and is the result of microstructural changes in the raw material in the cooking stage [19]. Understanding the effect of processing conditions on a desirable food is critical; therefore, the microstructure is important to understanding the process of water absorption in fried products [20]. In the same way, the study of the atmospheric or immersion frying process is important to understand mass transfer, particularly oil absorption in the finished product [21]; also, current consumer trends require lower fat contents but optimal sensory characteristics.

In addition, vacuum frying has emerged as an alternative to preserve some nutritional parameters [22, 23]; improving the quality attributes of fried products, due to the process being carried out at subatmospheric pressure, implies lower operating temperatures than conventional frying [24]. An important difference between vacuum frying and deep-frying is the lower water boiling point, because, at lower pressures, lower frying temperatures can be used, which impacts product quality [25]. Research on the frying process in native starchy matrices is very limited; therefore, this research focuses on evaluating the thermophysical, property sensory quality, and microstructure of traditionally prepared Colombian coastal Carimañola under atmospheric and vacuum frying conditions.

## 2. Materials and Methods

### 2.1. Raw Material

Cassava (*Manihot esculenta*) variety MCOL 2215, bought at the central distribution center in Cartagena, Bolívar (Colombia), was used to make the coastal Carimañola samples. Refined palm oil was used, which was purchased in a local supermarket, along with ground beef for the filling.

### 2.2. Carimañola Elaboration Process

The cassava samples were immersed in hot water at a controlled temperature using type J thermocouples. The initial temperature of the product was measured, and a thermocouple was placed in the water bath to measure the process temperature. In the initial stage of the experiment, the water bath temperature was controlled within ±1°C of the set point (99°C).

The water was not agitated, and, when the temperature in the center of the sample was in the range of 70-75°C for 15 minutes, the samples were removed [26]. Subsequently, the cooked cassava was ground in a Corona mill adapted to a mechanical system to make the dough. The Carimañolas were made with cassava flour dough, and the dough was molded into a concave shape to be filled with ground beef; 60 g of dough and 10 g of meat were used for the Carimañolas.

### 2.3. Vacuum Frying Conditions

The vacuum frying was done with a GASTROVAC®, 40∗26∗46 cm, with a maximum capacity of 10.5 liters and 220 V [26]. The maximum vacuum pressure was 30 kPa, at which the boiling temperature of water is 70°C. Three deltas ΔT1 = 50°C, ΔT2 = 60°C, and ΔT3 = 70°C were used to define the temperature of the frying process. Therefore, the oil temperatures employed were 120°C, 130°C, and 140°C. The frying times employed ranged from 180 to 300 seconds, which were established by preliminary tests. To start the process, the Carimañolas were placed in a metal basket attached to the lid of the fryer, the fryer was closed, and when the desired pressure was stable, the knob was lowered to immerse the basket in the hot oil and to start the frying process. After the frying time, the basket was removed from the oil, the vacuum chamber was opened, and the hose was disconnected to balance the atmospheric pressure. Afterwards, the product was removed from the equipment.

### 2.4. Atmospheric Frying Conditions

Palm oil was used as the heat transfer medium in a 6 L, electric, stainless steel fryer, with the temperature controlled with a thermostat (±0.1°C precision) coupled to a data acquisition system, similar to the study by [27]. The temperatures used were approximately 150°C, 160°C, and 170°C, for times between 155 s and 325 s. First, the oil was heated to the selected temperature; then, the samples were immersed in the oil for the established time. Subsequently, the samples were removed from the oil, allowed to cool for five minutes in a desiccator, and placed in airtight bags, and then, the samples were analyzed. The frying of each treatment was carried out in triplicate.

### 2.5. Bromatological Characterization

The bromatological analysis was carried out on samples of 7 Carimañolas, one sample before the frying process, 3 fried under vacuum conditions (temperatures of 120, 130, and 140°C and time of 240 s), and 3 samples fried under atmospheric conditions (temperatures of 150, 160, and 170°C and time of 240 s). The methodology proposed by the Official Association of Analytical Chemists was followed to carry out the following determinations: moisture, protein, fat, ash [28], and fiber. All samples were macerated before each test to obtain homogeneous samples. In addition, following equations (1) and (2), total carbohydrates and the amount of energy (calories) were calculated, respectively,

(1) $$\begin{array}{c} \%\text{carbohydrates}=100-\left(\%\text{moisture}-\%\text{protein}-\%\text{fat}-\%\text{ash}-\%\text{fiber}\right), \end{array}$$

(2) $$\begin{array}{c} \text{Calories }\left(\text{kcal}/100\text{ gr}\right)=\left(4*\%\text{protein}\right)+\left(9*\%\text{fat}\right)+\left(4*\%\text{carbohydrates}\right). \end{array}$$

### 2.6. Determination of Thermophysical Properties

The specific heat (Cp.), density (*ρ*), conductivity (*k*), and thermal diffusivity (*α*) of Colombian coastal Carimañola samples fried at temperatures of 120, 130, and 140°C (vacuum frying) and 150, 160, and 170°C (atmospheric frying) during the same frying time of 240 were determined using the computational program named CTCIA (Heat Transfer Coefficients in Food Engineering) developed by Tirado et al. [29]. This program is based on the formulas suggested by Alvis et al. [30] for the calculation of each of the thermophysical properties as a function of product composition and frying temperatures.

### 2.7. Experimental Design and Statistical Analysis

The process of atmospheric and vacuum (30 kPa) frying of the Carimañolas was developed using a nonrandomized rotatable compound central design (CCD-R), conformed by a factorial fraction (2*k* = 2^2^ = 4) and increased by 4 axial points (*α*) and 5 central points, for a total of 13 experimental runs for each frying process. Equation (3) was used to calculate the number of experimental runs for each type of frying:

(3) $$\begin{array}{c} N=2^{k}+2k+n_{0}, \end{array}$$

where *N* represents the total number of experimental runs, 2*k* is axial points, *n*_0_ is central points, and *k* is the number of factors. The low and high levels of the vacuum frying process factors (*X*_1_ = temperature (°C) and *X*_2_ = time (s)) were set by preliminary tests and by literature references. On the other hand, the lower and upper limits of the design were chosen considering the number of factorial points; therefore, *α* = (4)^1/4^ = 1.4142 and were coded as shown in Tables 1 and 2. The experimental data of the response variables were fitted to the second-order regression models, equation (4), using the least-squares method.

An analysis of variance (bifactorial ANOVA) was used to identify statistical differences between the data of the response variables. The means were compared using Tukey's HSD test with a significance level of 5% (*P* ≤ 0.05). The data were analyzed in the statistical program Statgraphics Centurion 16.1.15 (Corporation, U.S.A.):

(4) $$\begin{array}{c} Y=\beta_{0}+\beta_{1}X_{1}+\beta_{2}X_{2}+\beta_{12}X_{1}X_{2}+\beta_{11}X_{1}^{2}+\beta_{22}X_{2}^{2}+\varepsilon . \end{array}$$

### 2.8. Microstructure

The microstructural changes of two Carimañolas, one fried under atmospheric conditions (170°C and 240 s) and one vacuum fried (140°C and 240 s), were observed with a scanning electron microscope (XL from Phenom-World, USA) with an acceleration potential of 15 kV. The samples were immersed in petroleum ether for 4 hours to remove the oil from the surface. Finally, the samples were placed on aluminum beads, coated with gold under vacuum conditions, and observed in the scanning electron microscope (SEM). The microscope had a secondary silicon detector (SSD) and a material analysis system (LN2 free), Beryllium (Be): 0.5 mil (12.5 *μ*), which provided the electronic micrograph [31, 32].

### 2.9. Sensory Evaluation: Profile Test

To evaluate the sensory quality of the Carimañolas, optimized treatments were chosen according to moisture content and oil adsorption for each type of frying, and a comparison was made of a Carimañola prepared by atmospheric frying (170°C and 240 s) and vacuum frying (140°C and 240 s). A five-point hedonic scale was used to individually evaluate the parameters of color, odor, flavor, crispness, and fatty sensation. The scale points ranged from “I like it very much” to “I don't like it very much.” An untrained panel of 60 tasters was used for this study.

## 3. Results and Discussion

### 3.1. Bromatological Analysis

The bromatological characteristics by type of frying of the coastal Carimañola samples are described in Table 3; one sample was not fried, and the others were fried under vacuum and atmospheric conditions at a fixed time of 240 s. The results show that for some elements or macronutrients, the values do not follow a clear trend of increase or decrease with frying temperatures.

The moisture content of unfried Carimañola was 65.60%, which decreased with increasing temperature, showing statistically significant differences (*P* ≤ 0.05) between the samples obtained by atmospheric and vacuum frying. Oyedeji et al. [33] reported a similar initial moisture content for cassava slices, which was 64%; the authors explained that the moisture content in the product decreased with increasing temperatures, justifying this trend to the fact that as the frying process progressed, the rate of moisture removal was reduced by the elimination of a greater amount of free moisture in the samples. The results observed in this research are similar to those of [34] in their study on mass transfer during vacuum frying of Malanga discs, who reported that it was evident that moisture decreased as temperature increased. This is evidenced in the study of the properties of sweet potatoes fried under atmospheric conditions, carried out by [30], where the moisture content of samples fried at 150°C presented higher moisture content than those subjected to temperatures of 170°C and these in turn than those at 190°C. Table 3 also shows that moisture contents are higher in samples processed under atmospheric conditions than in those processed under vacuum, due to the fact that the reduction in pressure causes a decrease in the boiling point, thus evaporating the water contained in the food at a temperature lower than 100° [35]. In addition, it has been reported that protein denaturation during frying also contributes to the evaporation of water trapped within the protein structures [36].

Oil absorption is a phenomenon that determines the extent of crust formation and the volume available for oil infiltration [37], which in the vacuum frying process is produced during periods of pressurization and cooling [38]. Table 3 shows that both vacuum frying and conventional or atmospheric frying cause oil absorption to decrease with increasing temperature. Esan et al. [39] in their study on process optimization by response surface and quality attributes of vacuum fried sweet potato chips found that increasing frying temperature and vacuum pressure reduces the absorbed oil content at constant frying time; the authors indicate that this behavior can be attributed to the effect resulting from using lower thermal driving force at the lower temperature to remove water from the products. [40] reported that apple slices fried under atmospheric conditions after a certain frying time absorb a higher percentage of oil than slices fried under vacuum; the authors explain that the increase in driving force increases the difference in oil absorption between vacuum frying and atmospheric frying increases. In addition, they explain that the lower temperatures involved in vacuum frying of foods cause fewer structural changes (degradation of tissues/constituents) leading to lower oil absorption. Moreover, [41] observed that the percentage of oil absorption of vacuum fried empanadas was higher when low temperatures were used, due to the replacement in the food of oil by the water evaporated in the frying process.

A reduction in protein content was found in the Carimañolas during the frying process; this is due to the fact that proteins denature when exposed to temperatures above 65°C, and consequently, their biological value decreases drastically [42, 43]. Table 3 shows that the percentage of proteins was higher with the increase of the frying temperature for the types of frying; these results agree with what was indicated by [44, 45] who reported that as the temperature increased, the protein increased; the authors justified that the result is due to the loss of humidity of the product; and this causes a concentration of solutes. The samples processed through conventional frying reached lower protein values, which is a consequence of a lower concentration of solutes in the food due to the lower loss of moisture. [46] reported a similar 5.37% protein content in Oca chips.

Regarding the carbohydrate content in Carimañolas, the increase of this macronutrient with the increase of oil temperature is significant. Some authors explain that this trend is due to the fact that carbohydrates are concentrated in the product by the evaporation of water in the frying process [47], which is reasonable when analyzing the kinetics of moisture loss of coastal Carimañola in both types of frying; this means that for a time of 240 s, there is greater moisture loss when the temperature increases. At higher temperatures, a greater evaporation rate can be noted, as in the study of [48], who calculated a higher level of water evaporation at 185°C than at 145 or 165°C. Similar results were reported by [49]; in their study about the evaluation of the final quality of arepa with egg obtained by deep-frying, the author found that the percentage of carbohydrates increased at higher temperature levels, whose values were 19.35, 28.76, and 35.53 at temperatures of 170, 180, and 190°C.

### 3.2. Thermophysical Properties

Thermophysical properties directly influence the heat transfer of food; therefore, it is important to study them for accurate calculation in equipment design, to contribute to packaging selection [50], and to improve product quality. These, in turn, depend on factors such as temperature applied in heat treatment and the chemical composition of the food [14, 51].  Table 4 shows the values of conductivity, specific heat, density, and thermal diffusivity of coastal Carimañola fried in vacuum conditions at temperatures of 120, 130, and 140°C and the Carimañola fried in atmospheric conditions at temperatures of 150, 160, and 170°C at a fixed time of 240 s and one control sample (without frying).

The values for conductivity and specific heat in the samples of Colombian coastal Carimañolas prepared under vacuum pressure showed a decrease as the process temperature increased, which could be due to the reduction of the moisture content present in the food. However, in samples fried under atmospheric conditions, conductivity increased at a temperature of 160°C and then decreased at a temperature of 170°C. In a study carried out by [30] on the thermophysical properties of sweet potato pieces fried in atmospheric conditions at temperatures of 150, 170, and 190°C, they reported that the thermal conductivity and specific heat presented higher values at 150°C; these authors associated this behavior to the moisture content of the food, which decreases during the frying process; therefore, it can be stated that these properties do not remain constant throughout the process.

Conductivity can be defined as the speed at which heat flows in a material influenced by a temperature gradient. In this sense, conductivity in foods, which are generally nonhomogeneous substances, depends on their composition, structure, and any factor affecting porosity, pore arrangement, density, temperature, and moisture content and is also affected by the presence of voids in the food and the degree of structural homogeneity [52, 53]. It was found that the highest thermal conductivity values were recorded in the samples of Carimañolas prepared under vacuum pressure, at temperatures of 120, 130, and 140°C, corresponding to 0.477, 0.473, and 0.470 W/m °C, respectively. In addition, the lowest thermal conductivity value was recorded at 170°C which is 0.457 W/m °C; this was the highest temperature at atmospheric pressure. There is a close relationship between the thermal conductivity of foods and their moisture content, since moisture plays an important role in transport properties, especially in those that exchange mass and energy. It has been reported that the thermal conductivity of potatoes with a drying process decreased with decreasing moisture content [54].

Other researchers [55] reported that thermal conductivity and thermal diffusivity presented an increase proportional to the temperature during the deep fat frying of arepa with egg; in addition, they affirmed that these properties depend on the microstructure and the constituents of the food; furthermore, they relate it to the moisture content of the food, because when this content is decreased by the frying process, it causes a concentration of the solids present facilitating the diffusion of heat. The diffusivity behavior observed for Carimañolas corresponds to that reported by [56], which explains that the thermal diffusivity of Carimañolas increases with temperature, which may be due to the fact that the concentration of solids due to the increase in temperature implies a greater diffusion of heat.

### 3.3. Microstructure

**Figure 1:**
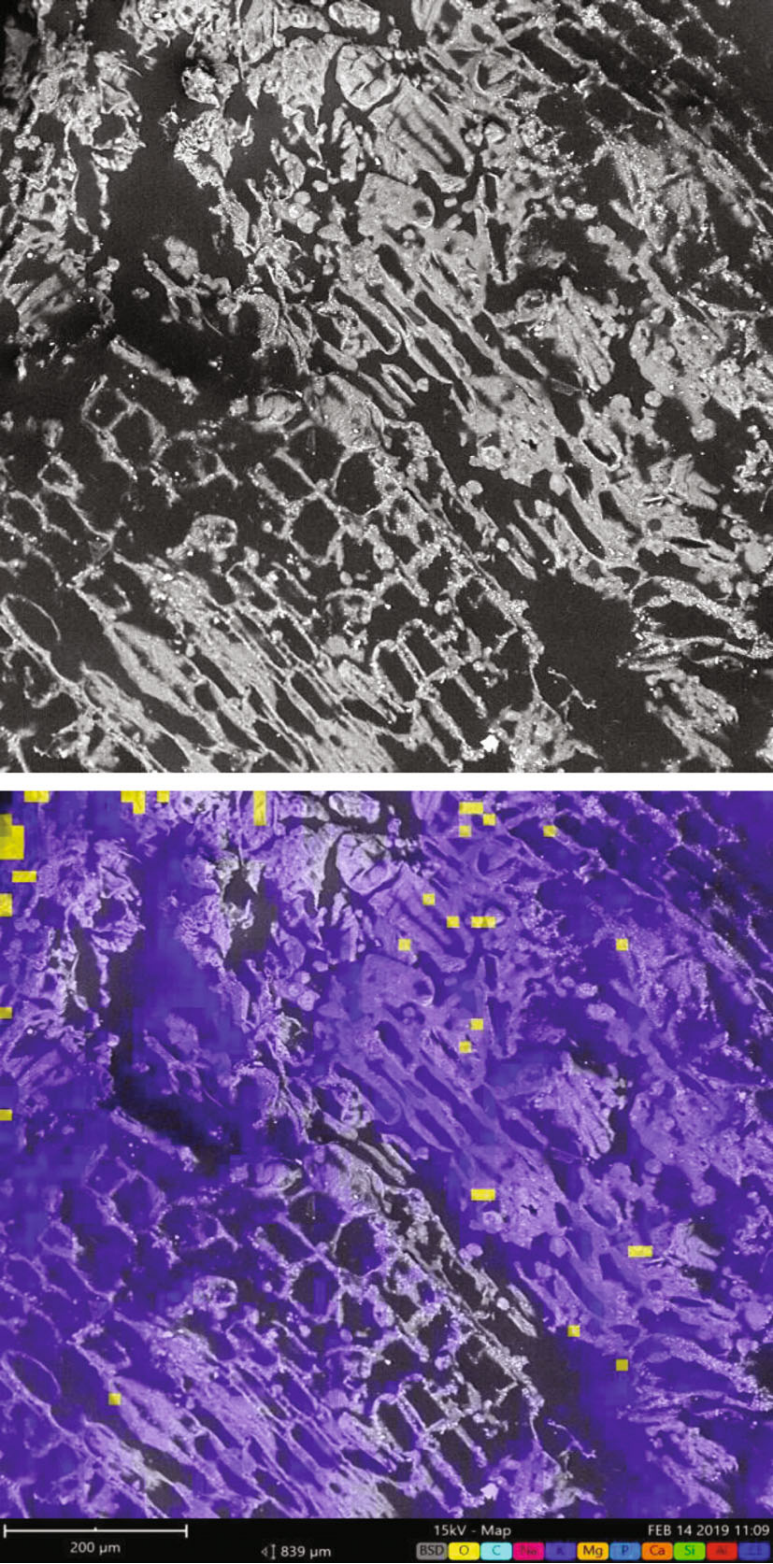
Micrograph of Colombian coastal Carimañolas obtained by vacuum frying.

**Figure 2:**
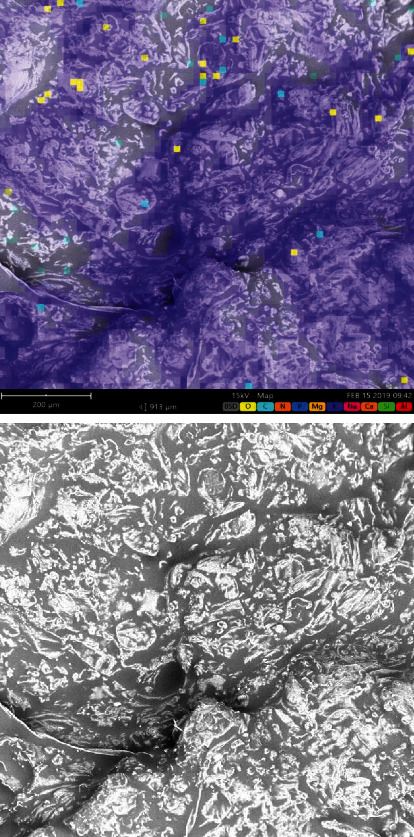
Micrograph of Colombian coastal Carimañolas obtained by atmospheric frying.

The micrograph for the vacuum frying (see Figure 1) reflected a much more formed structure than for the Carimañolas fried with immersion (see Figure 2). This phenomenon has been reported in various food matrices. Martínez-Pantoja et al. [57] who studied banana chips indicated that the frying process entails a contraction of matter, with a consequent loss of moisture [58] that results in a hardening of the crust structure and low maximum breaking force values.

Other researchers have shown that the cellular structure of a food matrix is affected during frying, such as Wang et al. [59]. In this sense, the temperature used in the process influences the texture of the food, forming larger or smaller structures. Chitrakar et al. [60] who studied vacuum fried Chinese yams found a more fractured cellular structure with microscopic channels as the result of the rapid evaporation of moisture. Damage at the cellular level can affect other critical properties such as the rate of moisture removal and oil absorption.

Cocio Pulgar [61] studied potato chips and reported that the deterioration generated by the outflow of water in the form of water vapor altered the microstructure of the samples because the cells that were not cut in the production process swelled because of the accumulation of water vapor in the dehydration stage until rupturing, with subsequent toasting and hardening of the broken cell wall.

It has been reported that the microstructure is affected by high temperatures in similar starch matrices, influencing quality parameters in products and significantly affecting the final structure of the food. Samples fried under vacuum conditions can have a lower degree of deterioration because the process temperatures are generally lower than in atmospheric frying [62]. The micrograph of the Carimañola samples made with atmospheric frying (see Figure 2) showed that part of the starch was destroyed, and consequently, a more deformed structure was observed. Chen et al. [63] explained that, during the frying stage, starch can dry out quickly because the temperature is higher than the boiling point of water; therefore, continuous evaporation of water favors partial gelatinization of the starch.

The samples obtained with vacuum frying (see Figure 1) maintained a net-like structure, with square openings. Yang et al. [20] who studied fresh and prefrozen potato strips reported that bleached samples had regular, polygonal holes that lost this shape when fried because of starch gelatinization. Similarly, [64] observed that samples obtained with conventional frying had more damaged cells with deep and large grooves, but the diameter of their pores was significantly greater, which could explain the higher oil absorption rate and greater crispiness in the fried samples.

On the other hand, Figure 2 shows greater luminosity in the micrograph, explained by a high oil content in the atmospheric frying Carimañola samples. The phenomenon of oil absorption has been studied by [65], who reported that deep-frying had a higher oil content in the final samples. The luminosity reflected in that study evidenced a significant oil concentration. Different luminosity levels indicate the distribution of oil in analyzed samples, where a blue hue indicates the density of the weakest proton signal [66]. Different authors have reported that the size of pores in samples is greater when the frying temperature is increased, which causes a high absorption of oil and the flow of water vapor and oil. Pressure affects porosity, pore number and size, and microstructure in food [67].

### 3.4. Sensorial Evaluation

**Figure 3:**
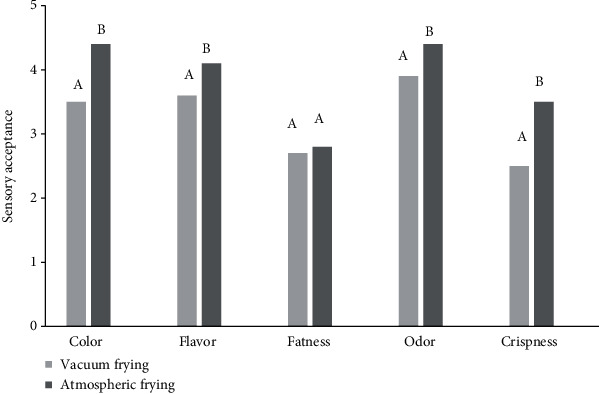
Samples of Colombian coastal Carimañolas fried under vacuum and atmospheric conditions. Means within a bar followed by the same letter are not significantly different (*P* ≤ 0.05).

The results of the sensory analysis of the Carimañolas made with atmospheric and vacuum frying are shown in the following radial graphs (see Figure 3), which compared the level of acceptability of each parameter. Both samples presented a good level of general acceptance. The parameters with greater qualification included odor, taste, and color. For the vacuum fried samples, 53.33% of the panelists rated the smell as “I like,” and 33.33% indicated the same for taste. In the comments, the panelists indicated that this sample better conserved the characteristics of cassava. However, the sample made with conventional frying obtained 56.67% for “I like it a lot” in the odor attribute, 50% for color, and 66.67% for flavor. It should be noted that Carimañola is a traditional food and, consequently, has specific sensory characteristics that consumers prefer.

On the other hand, when comparing the results between both frying methods, it was evident that vacuum frying provides sensory characteristics that are accepted by consumers [68], presenting an alternative for making fried products. Alvis et al. [69] stated that high temperatures, such as those used in atmospheric frying, result in mechanical and sensory properties that are palatable to consumers, such as color, flavor, and texture.

The main disadvantage of vacuum frying was evidenced in the crunchiness; some authors have indicated that the development of a crunchy texture is related to the heat transfer rate. Conventional frying generates this characteristic with a high heat transfer rate seen in the high oil temperatures [19].

## 4. Conclusions

The frying process is a crucial stage for obtaining fried products. This study demonstrated the effect of high temperatures on atmospheric frying on the microstructure of the samples. The bromatological composition of both samples showed that vacuum frying allows maintaining some macronutrients of the food; furthermore, a higher fat gain was reported in atmospheric frying, which is one of the main attributes to be controlled in foods. On the other hand, the study of the thermophysical properties showed that thermal diffusivity increased with temperature. The micrographs indicated that the vacuum fried Carimañola samples maintained the final structure a little longer because of the lower temperatures applied with this technique. Consequently, the reduction of temperatures in vacuum frying could better preserve the initial quality of raw materials, in this case, cassava. The sensory evaluation demonstrated good acceptability for the analyzed attributes. It was concluded that the vacuum frying technique is an alternative for processing and making Carimañolas with optimal quality and acceptable characteristics.

## Figures and Tables

**Table 1:** Rotational composite central design matrix (CCD-R) with axial points and coded center points: vacuum frying.

Variables	Encoder symbol	Coded levels				
		*α*1 = −1.4241 (axial)	-1 (low)	0 (medium)	+1 (high)	*α*2 = +1.4241 (axial)
Temperature (°C)	*X*_1_	115.858	120	130	140	144.142
Time (s)	*X*_2_	155.26	180	240	300	324.85

**Table 2:** Rotational composite central design matrix (CCD-R) with axial points and coded center points: atmospheric frying.

Variables	Encoder symbol	Coded levels				
		*α*1 = −1.4241 (axial)	-1 (low)	0 (medium)	+1 (high)	*α*2 = +1.4241 (axial)
Temperature (°C)	*X*_1_	145.85	150	160	170	174.65
Time (s)	*X*_2_	155.26	180	240	300	324.85

**Table 3:** Bromatological composition of Carimañolas fried under vacuum and atmospheric conditions.

	*T* (°C)	Moisture (%)	Fat (%)	Ash (%)	Protein (%)	CHO (%)	Fiber (%)	Calories (kcal/100 g)
	Raw	65.60^a^	4.49^a^	0.92^a^	6.11^a^	20.59^ab^	2.29^a^	138.23^a^
Vacuum frying	120	60.95^c^	13.03^c^	0.92^a^	4.51^c^	18.32^b^	2.27^a^	182.53^b^
	130	58.14^d^	11.36^b^	0.94^c^	5.12^b^	22.16^a^	2.28^a^	188.64^b^
	140	57.52^d^	10.91^b^	0.92^a^	5.28^b^	23.00^a^	2.31^b^	189.73^b^

Atmospheric frying	150	63.75^b^	16.80^d^	0.92^a^	4.27^c^	11.94^d^	2.32^b^	216.04^d^
	160	62.87^b^	13.73^c^	0.93^b^	4.89^bc^	15.24^c^	2.34^c^	204.09^c^
	170	61.75^bc^	12.18^bc^	0.92^a^	5.06^b^	17.74^c^	2.36^c^	200.82^c^

Means within a column followed by the same letter are not significantly different (*P* ≤ 0.05).

**Table 4:** Thermophysical properties of Carimañolas fried under vacuum and atmospheric conditions.

	*T* (°C)	*k* (W/m°C)	Cp (J/kg °C)	*ρ* (kg/m^3^)	*α* (m^2^/s)
	Raw (25°C)	0.479693^a^	3334.4595^a^	1104.2496^a^	1.289 × 10^−7^^a^
Vacuum frying	120	0.477197^a^	3238.0394^b^	1030.2494^b^	1.430 × 10^−7^^b^
	130	0.472616^a^	3157.4513^c^	1044.3870^b^	1.433 × 10^−7^^b^
	140	0.470051^a^	3131.6040^c^	1042.1158^b^	1.440 × 10^−7^^b^

Atmospheric frying	150	0.465294^b^	3291.5021^ab^	974.4920^c^	1.426 × 10^−7^^b^
	160	0.463979^b^	3251.8666^b^	982.8640^c^	1.456 × 10^−7^^bc^
	170	0.457345^c^	3209.9494^b^	985.0489^c^	1.467 × 10^−7^^c^

Means within a column followed by the same letter are no significantly different (*P* ≤ 0.05).

## Data Availability

ZThe data used to support the findings of this study are available from the corresponding author upon request.
